# P Wave Indices—Advancing Our Understanding of Atrial Fibrillation-Related Cardiovascular Outcomes

**DOI:** 10.3389/fcvm.2019.00053

**Published:** 2019-05-03

**Authors:** Lin Y. Chen, Elsayed Z. Soliman

**Affiliations:** ^1^Cardiovascular Division, Department of Medicine, University of Minnesota Medical School, Minneapolis, MN, United States; ^2^Department of Epidemiology and Prevention, Wake Forest University Health Sciences, Winston-Salem, NC, United States

**Keywords:** atrial fibirillation, stroke, P wave indices, P wave duration, P wave axis

## Abstract

Atrial fibrillation (AF) is associated with an increased risk of ischemic stroke, heart failure, cognitive decline, dementia, myocardial infarction, sudden cardiac death (SCD), and all-cause death. Although these associations are firmly established, our understanding of the underlying mechanisms remains incomplete. Accumulating evidence suggests that left atrial (LA) abnormality or atrial cardiomyopathy may explain the relationship of AF to the aforementioned outcomes. P-wave indices (PWIs) reflect underlying atrial remodeling. In this mini review, we define representative PWIs, discuss state-of-the-art knowledge on the relationship between abnormal PWIs and AF-related cardiovascular outcomes (focusing on ischemic stroke and sudden cardiac death), and propose directions for future research. Our ultimate goal is to present a practical way forward to advance the emerging field of LA abnormality or atrial cardiomyopathy.

Atrial fibrillation (AF) is the most common sustained cardiac arrhythmia, and its prevalence is increasing over time (1, 2). AF is associated with an increased risk of ischemic stroke (3), heart failure (4), cognitive decline (5, 6), dementia (6), myocardial infarction (7), sudden cardiac death (SCD) (8–10), and all-cause death (8, 11, 12). Although these associations are firmly established, our understanding of the underlying mechanisms remains incomplete. The AF-stasis hypothesis is a widely accepted mechanism for AF-related thromboembolism (13); however, recent data compel us to re-evaluate this hypothesis: Novel insights into the lack of temporal relationship between AF episodes and ischemic stroke events in AF patients (14, 15), and data linking markers of left atrial (LA) abnormality or atrial cardiomyopathy with ischemic stroke in the absence of AF (16, 17), suggest that some entity—other than the irregular rhythm of AF—may be driving the risk of thromboembolism.

LA abnormality or atrial cardiomyopathy is an entity that encompasses alterations in macro- and micro-structure; reservoir, conduit, and contractile function; and electrical conduction in the atria (18). Molecular changes involving endothelial function, coagulation, inflammation, oxidative stress, and other pathways also fit into this paradigm (18). Methods to detect these alterations are diverse—such as magnetic resonance imaging, 3D-echocardiogram, body surface mapping, etc.—inconclusive, and are limited by technical challenges in implementation and interpretation, low acceptability by patients, and high cost (19). Other methods include measurement of electromechanical delay by tissue Doppler echocardiography (PA-TDI), which is an emerging parameter to measure left atrial fibrosis (20). Although there is a pressing need to better characterize LA abnormality or atrial cardiomyopathy to improve our ability to predict AF-related outcomes, currently available tools remain firmly in the realm of research, and are not ready to be used clinically at the bedside.

Alterations in atrial activation measured through analysis of P-wave morphology—P-wave indices (PWIs)—has been associated with atrial remodeling (21–23) and ischemic stroke (24–26). PWIs are also associated with increased risk of AF (27–29). In individuals with non-permanent AF, periods of sinus rhythm present an opportunity to detect underlying atrial cardiomyopathy through measurement of PWIs. These PWIs include P-wave axis, P-wave duration, advanced inter-atrial block (aIAB), and P-wave terminal force in lead V1 (PTFV1), and others. In this mini review, we define representative PWIs, discuss state-of-the-art knowledge on the relationship between abnormal PWIs and AF-related cardiovascular outcomes (focusing on ischemic stroke and SCD), and propose directions for future research. Our ultimate goal is to present a practical way forward to advance the emerging field of LA abnormality or atrial cardiomyopathy.

## P Wave Indices

PWIs include P-wave axis, P-wave duration (maximum, minimum, and mean), aIAB, PTFV1, P-wave area (maximum, minimum, and mean), P-wave dispersion, signal average P-wave, and others. Of all PWIs, P-wave axis is the only one that is routinely reported on all standard 12-lead ECGs. Calculation of other PWIs requires ECG digitalization and specialized analytic software to allow for precise measurements making them impractical for everyday clinical use, particularly in the primary care setting. Therefore, compared with other PWIs, P-wave axis holds the distinct advantage of being readily available at the bedside for clinical use. In this review, we will focus on four PWIs that have been extensively reported in the literature: P-wave axis, P-wave duration, aIAB, and PTFV1.

### P-Wave Axis

**Figure 1:**
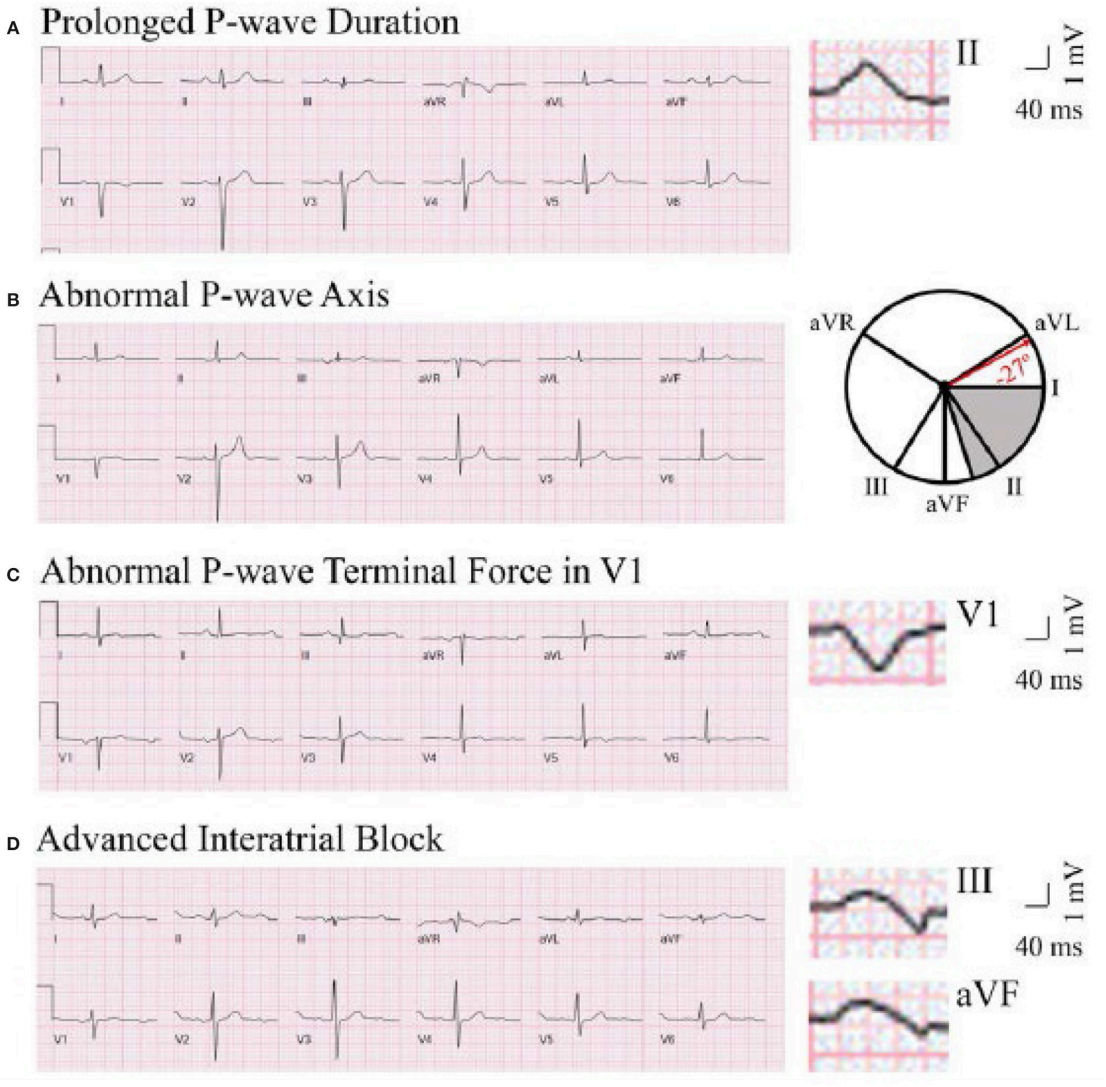
Representative ECG tracings of abnormal P-wave indices. A through **(D)**, Prolonged P-wave duration **(A)**, abnormal P-wave axis **(B)**, abnormal P-wave terminal force in V1 **(C)**, and advanced interatrial block **(D)**. **(A)** The maximal P-wave duration is seen in lead II (136 ms). **(B)** The gray area on the hexaxial reference system (lead I 0°, lead II 60°, aVF 90°, aVR −150°, aVL −30°) represents normal P-wave axis (0–75°). The P-wave axis on B is −27°. **(C)** The P-wave terminal force is −9,632 μV*ms (amplitude −112 μV, duration 86 ms). **(D)** The maximal P-wave duration is seen in lead III (136 ms). Biphasic P-waves can be seen in III and aVF. This figure has been republished from Maheshwari et al. (30). Chen and Soliman are allowed to republish this figure, per American Heart Association Journal Policy.

P-wave axis is a measure of the net direction of atrial depolarization. It is determined by measuring net positive or negative P-wave deflections on all six limb leads and calculating the net direction of electrical activity using the hexaxial reference system. Abnormal P-wave axis is defined as any value outside 0–75° (Figure 1) (31).

### P-Wave Duration

P-wave duration is a reflection of the time required for right and left atrial depolarization. It is measured from the conclusion of the T-P segment (P wave onset) to return to baseline (PR interval). For biphasic P-waves, P-wave duration encompasses both positive and negative deflections from baseline. Prolonged P-wave duration is present if the maximum P-wave duration in any lead is >120 ms on a standard 12-lead ECG (Figure 1) (32, 33).

### Advanced Inter-Atrial Block

aIAB is an indicator of inter-atrial conduction block in the Bachman's bundle such that the LA is activated superiorly. It is defined as prolonged P-wave duration + biphasic P-wave morphology in leads III and aVF with biphasic morphology or notched morphology in lead II (Figure 1) (32).

### P-Wave Terminal Force in Lead V1

PTFV1 is a measure of LA activation. It is computed by multiplying the duration (ms) and the depth (μV) of the downward deflection (terminal portion) of the P-wave in lead V1. Abnormal PTFV1 is defined as ≤ -4,000 μV^*^ms (Figure 1) (33).

The difference between aIAB and prolonged P-wave duration seen in LA enlargement is worth noting. The key difference between advanced interatrial block (aIAB) and enlarged left atrium (LA) is the direction of the activation from right atrium (RA) to LA. In aIAB, the marked delay of activation from RA to LA across the blocked Bachmann Bundle forces the impulses in the RA to be directed toward the atrioventricular node first before progressing caudocephalically to depolarize the LA. This results in biphasic (typically positive-negative phases) and wide P waves in leads II, III, and aVF (34).

On the hand, in LA enlargement, LA depolarization is initiated in normal direction on arrival of the electrical wave at the atrial septum through Bachmann Bundle. However, depolarization takes a longer time within the LA due to enlargement. The delayed activation in LA due to larger size only leads to prolonged P wave duration without the positive-negative phases that are seen in aIAB. The notched P wave that is sometimes observed in enlarged LA occur as a result of the overlap between the depolarization of the RA and LA due to the longer time the LA takes in completing its activation (34).

## P Wave Indices and Ischemic Stroke

In recent years, several epidemiological studies have implicated abnormal PWIs as independent risk factors for ischemic stroke (Table 1). Some of these studies indicate a stronger association with cardioembolic than thrombotic stroke (24) or with non-lacunar than lacunar stroke (17, 24), consistent with the notion that PWIs are a marker of abnormal atrial structure and function which promote thrombosis and subsequent embolism.

**Table 1:** Selected studies relating abnormal P-wave indices with risk of ischemic stroke.

**References**	**P-wave indices**	**Outcome**	**Atrial fibrillation**	**Adjusted effect estimates**
Kamel et al. (16)	PTFV1 P-wave area P-wave duration	Incident ischemic stroke	Excluded and adjusted	PTFV1: HR per 1-SD increase, 1.21; 95% CI, 1.02–1.44. No associations with P-wave area and P-wave duration.
Kamel et al. (35)	PTFV1 P-wave area P-wave duration	Prevalent and incident brain infarcts on MRI	Excluded and adjusted	PTFV1: Associated with prevalent brain infarcts (RR per SD, 1.09; 95% CI, 1.04–1.16) but not with incident brain infarcts. No associations with P-wave area and P-wave duration.
Kamel et al. (17)	PTFV1	Incident ischemic stroke subtypes	Adjusted	Associated with incident non-lacunar stroke (HR, 1.49; 95% CI: 1.07–2.07) but not with lacunar stroke.
O'Neal et al. (25)	aIAB	Incident ischemic stroke	Adjusted	HR, 1.63; 95% CI, 1.13–2.34.
Maheshwari et al. (24)	P-wave axis	Incident ischemic stroke subtypes	Adjusted	Ischemic stroke: HR, 1.50; 95% CI, 1.22–1.85. Cardioembolic stroke: HR, 2.04; 95% CI, 1.42–2.95. Thrombotic stroke: HR, 1.32; 95% CI, 1.03–1.71.
Maheshwari et al. (30)	PTFV1 P-wave axis P-wave duration aIAB	Incident ischemic stroke	Included	P-wave axis: HR, 1.88; 95% CI, 1.36–2.61. aIAB: HR, 2.93; 95% CI, 1.78–4.81. No associations with PTFV1 and P-wave duration Only P-wave axis resulted in significant improvement in C-statistic and improvement in risk classification of ischemic stroke compared with CHA_2_DS_2_-VASc.

A recent study by Maheshwari et al. (30) went one step further to determine whether consideration of PWIs would improve ischemic stroke prediction in individuals with AF, above and beyond the current paradigm, which is the CHA_2_DS_2_-VASc (congestive heart failure, hypertension, age, diabetes, stroke, vascular disease, sex) score (36). Despite its many limitations (37, 38), the CHA_2_DS_2_-VASc is the most widely used scoring system to assess the risk of ischemic stroke and to determine the need for anticoagulation in people with AF. Using data from two prospective community-based cohort studies—Atherosclerosis Risk in Communities (ARIC) and Multi-Ethnic Study of Atherosclerosis (MESA)—Maheshwari et al. found that of the PWIs considered (P-wave axis, P-wave duration, aIAB, and PTFV1), abnormal P-wave axis was the only PWI associated with increased ischemic stroke risk independent of CHA_2_DS_2_-VASc variables, and that resulted in meaningful improvement in stroke prediction. The β estimate of abnormal P-wave axis in the regression model was approximately twice that of the CHA_2_DS_2_-VASc variables, and thus abnormal P-wave axis was assigned 2 points to create the P_2_-CHA_2_DS_2_-VASc score. Compared with the CHA_2_DS_2_-VASc score, the P_2_-CHA_2_DS_2_-VASc score improved the C-statistic (95% CI) from 0.60 (0.51–0.69) to 0.67 (0.60–0.75) in ARIC and 0.68 (0.52–0.84) to 0.75 (0.60–0.91) in MESA (validation cohort). In ARIC and MESA, the categorical net reclassification improvements (95% CI) were 0.25 (0.13–0.39) and 0.51 (0.18–0.86), respectively, and the relative integrated discrimination improvement (95% CI) were 1.19 (0.96–1.44) and 0.82 (0.36–1.39), respectively.

Therefore, we now have some evidence to suggest that PWIs are not only associated with AF-related ischemic stroke, but they can also improve risk classification of ischemic stroke in people with AF. Before these PWIs—particularly abnormal P-wave axis—can be routinely used at the bedside, remaining knowledge gaps will need to be addressed as outlined in the final section of this review. In addition, some limitations of PWIs will need to be considered. For example, PWIs cannot be measured in patients with permanent AF.

## P Wave Indices and Sudden Cardiac Death

AF is associated with a 2.5-fold increased risk of SCD (8). Risk factors for SCD in people with AF include higher age, higher body mass index, coronary heart disease, hypertension, diabetes, cigarette smoking, left ventricular hypertrophy, higher heart rate, and lower albumin (39). Recent evidence also suggests that PWIs are independently associated with elevated risk of SCD.

Tereshchenko et al. (40) evaluated a simplified ECG metric of abnormal PTFV1—deep terminal negativity of P wave in V1 (DTNPV1)—in relation to SCD in ARIC. DTNPV1 was defined from the resting 12-lead ECG as presence of biphasic P wave (positive/negative) in V1 with the amplitude of the terminal negative phase >100 μV. DTNPV1 was associated with an increased risk of SCD after multivariable adjustment including age, sex, coronary heart disease, AF, stroke, and heart failure (hazard ration [HR], 2.49; 95% CI, 1.51–4.10). DTNPV1 also improved reclassification: additional 3.4% of individuals were appropriately reclassified into a higher SCD risk group, as compared with traditional coronary heart disease risk factors alone.

In another ARIC-based investigation, Maheshwari et al. evaluated the prospective association of prolonged P-wave duration with SCD (41). The multivariable HR (95% CI) of prolonged P-wave duration for SCD was 1.70 (1.31–2.20). This association was attenuated but remained significant after updating covariates (including AF) to the end of follow-up with a HR of 1.35 (1.04–1.76).

Hence, evidence is accumulating to suggest that abnormal PWIs are associated with increased SCD incidence. The mechanisms remain unclear and may including the following: First, despite adjustment for cardiovascular risk factors or conditions, the abnormal PWI-SCD association may still be partially explained by the development of coronary heart disease, heart failure, or AF. Second, abnormal PWIs may reflect an arrhythmogenic myocardial substrate in the atrium and ventricle. In fact, the degree of interstitial left ventricular fibrosis on cardiac magnetic resonance imaging has been reported to be linearly associated with both increasing P-wave duration and increasing negative PTFV1 (42). Certainly, fibrotic remodeling can represent a common substrate between atrial and ventricular arrhythmias and may, in part, explain the relationships between markers of LA abnormality or atrial cardiomyopathy and SCD.

## Future Directions and Conclusions

We propose additional investigation in the following areas to advance our understanding of AF-related cardiovascular outcomes.

- *Replication of currently know associations in other independent cohorts*: This is particularly important with respect to the P_2_-CHA_2_DS_2_-VASc score. Before this novel score can enter the realm of clinical medicine, its performance will need to be validated in diverse cohorts, including cohorts of people with different racial or ethnic groups.
- *Association with other AF-related outcomes:* Thus far, data on the relationship of PWIs to cognitive decline, dementia, or heart failure are lacking. More research on these outcomes is needed to confirm the prognostic value of PWIs in people with AF.
- *Biological correlation of abnormal PWIs:* The PRIMERI study reported that after adjustment for demographic characteristics, body mass index, maximum LA volume index, presence of scar, and left ventricular (LV) mass index, each decile increase in LV interstitial fibrosis was associated with 0.76 mV^*^ms increase in negative abnormal PTFV1 (95% CI, −1.42 to −0.09) and 5.4 ms prolongation of P-wave duration (95% CI, 0.9–10.0) (42). Maheshwari et al. found that abnormal P-wave axis and prolonged P-wave duration were both associated with lower LA ejection fraction (30). In addition, abnormal P-wave axis was associated with lower LA global longitudinal strain. More research is needed to confirm the relationship of abnormal PWIs to alterations in LA and LV macro- and micro-structure, systolic and diastolic function, and global electrical activation.
- *Role of anticoagulation for abnormal PWIs in the absence of AF:* AtRial Cardiopathy and Antithrombotic Drugs In Prevention After Cryptogenic Stroke (ARCADIA) (NCT03192215) is an NIH-funded randomized controlled trial (RCT) which tests the hypothesis that apixaban is superior to aspirin for the prevention of recurrent stroke in patients with cryptogenic ischemic stroke and atrial cardiomyopathy. The latter is defined by PTFV1 >5,000 μV^*^ms, LA size index ≥3.0 cm/m^2^ on echocardiogram, and serum amino terminal pro-B-type natriuretic peptide >250 pg/mL. RCTs focused on using oral anticoagulants for the primary prevention of ischemic stroke in people without AF but with abnormal PWIs will be the litmus test for the clinical utility of PWIs.

In conclusion, PWIs may emerge as a practical method to identify individuals at risk of adverse cardiovascular outcomes due to underlying LA abnormality or atrial cardiomyopathy. Before PWIs can change current clinical practice, more work will be needed to confirm their predictive value, elucidate their biological underpinnings, and ultimately, define the risks, and benefits of anticoagulation for abnormal PWIs to prevent ischemic stroke.

## Author Contributions

LC is the primary author and ES is the contributing author.

### Conflict of Interest Statement

The authors declare that the research was conducted in the absence of any commercial or financial relationships that could be construed as a potential conflict of interest.
